# Uniform Silica Coated Fluorescent Nanoparticles: Synthetic Method, Improved Light Stability and Application to Visualize Lymph Network Tracer

**DOI:** 10.1371/journal.pone.0013167

**Published:** 2010-10-18

**Authors:** Liman Cong, Motohiro Takeda, Yohei Hamanaka, Kohsuke Gonda, Mika Watanabe, Masutaka Kumasaka, Yoshio Kobayashi, Masaki Kobayashi, Noriaki Ohuchi

**Affiliations:** 1 Department of Nano-Medical Science, Graduate School of Medicine, Tohoku University, Sendai, Miyagi, Japan; 2 Department of Surgical Oncology, Graduate School of Medicine, Tohoku University, Sendai, Miyagi, Japan; 3 Department of Pathology, Tohoku University Hospital, Sendai, Miyagi, Japan; 4 Department of Electronics and Intelligent Systems, Tohoku Institute of Technology, Sendai, Miyagi, Japan; 5 Department of Biomolecular Functional Engineering, College of Engineering, Ibaraki University, Hitachi, Ibaraki, Japan; University of Helsinki, Finland

## Abstract

**Background:**

The sentinel lymph node biopsy (SLNB) was developed as a new modality in the surgical diagnosis of lymph node metastases. Dye and radioisotope are major tracers for the detection of sentinel lymph nodes (SLN). Dye tends to excessively infiltrate into the interstitium due to their small size (less than several nanometers), resulting in difficulties in maintaining clear surgical fields. Radioisotopes are available in limited number of hospitals. Fluorescent nanoparticles are good candidates for SLN tracer to solve these problems, as we can choose suitable particle size and fluorescence wavelength of near-infrared. However, the use of nanoparticles faces safety issues, and many attempts have been performed by giving insulating coats on nanoparticles. In addition, the preparation of the uniform insulating layer is important to decrease variations in the quality as an SLN tracer.

**Methodology/Principal Findings:**

We herein succeeded in coating fluorescent polystyrene nanoparticles of 40 nm with uniform silica layer of 13 nm by the modified Stöber method. The light stability of silica coated nanoparticles was 1.3-fold greater than noncoated nanoparticles. The popliteal lymph node could be visualized by the silica coated nanoparticles with injection in the rat feet.

**Conclusions/Significance:**

The silica coated nanoparticles in lymph nodes could be observed by transmission electron microscope, suggesting that our silica coating method is useful as a SLN tracer with highly precise distribution of nanoparticles in histological evaluation. We also demonstrated for the first time that a prolonged enhancement of SLN is caused by the phagocytosis of fluorescent nanoparticles by both macrophages and dendritic cells.

## Introduction

The metastatic status of the sentinel lymph nodes (SLN) is important for predicting the survival of malignancies. Since metastatic status of the SLN is highly predictive of involvement of the lymphatic system, the identification of SLN and biopsies are crucial in the staging of human cancers [1]–[3]. A tumor-negative SLN virtually excludes involvement of the regional lymphatics. Sentinel lymph node biopsy (SLNB) in breast cancer surgery has been developed to accurately assess axillary nodal status without removing most of the axillary contents, leading to avoid unnecessary axillary lymph node dissection in patients without axillary involvement. In clinical research, there are two major methods for detection of the SLN: the blue dye method and the radioisotopes method. However, there are disadvantages with each method. The dye method requires skill and the SLN cannot be identified without a skin incision. Moreover, as the diameter of dye particles is too small, it will diffuse and disappear in 15 to 20 min from the SLN, which restrict the duration of the procedure [4]. Furthermore, if the lymph node was embedded in fat tissue, then it may result in the negative or negligible detection of the lymph node [5]–[6]. The radioisotope method requires radioactive agents and therefore can only be performed at a limited number of hospitals because of the regulations for handling radioactive agents.

To make up for these disadvantages, we used fluorescent nanoparticles to efficiently visualize SLN from outside the body and determined that the appropriate size and fluorescence wavelength in detection of SLN in animals from previous studies [7]–[8].

Polymer nanoparticles incorporating fluorescent dyes have been used for a wide variety of applications [9]. They have the potential for use as novel biomarkers to make major advances in medical diagnostics, targeting therapeutics, molecular biology and cell biology as ultrasensitive tracers for multicolor labeling [10]–[12]. Among such applications, the long-lasting marking of SLN or malignant lesions during the cancer surgery has a definite advantage for the surgical treatment [13]–[14]. However, many problems still remain for SLNB, such as safety, techniques to control size of nanoparticles, ideal track time and ideal modalities for the histological detection of sentinel node metastases. Preparation of insulating layer with physically stable material is an appropriate solution for some of these problems and silica is a good candidate as such materials.

In our previous study we performed silica coating for the 100 nm particles and the silica coated nanoparticles exhibited a more stable fluorescence to laser irradiation than the noncoated ones [10]. We also indicated that the appropriate size for SLNB is around 40 nm [8]–[9]. There have been no previous attempts to prepare silica coated fluorescent nanoparticles of 20 and 40 nm. Since silica coating is physically stable, it is expected to reduce the toxicity and enable tissue processing for microscopic observation without breakup of nanoparticles. In the present study, we performed silica coating for fluorescent 20 and 40 nm nanoparticles utilizing a seeded polymerization technique based on the Stöber method and tried to clarify the suitability as SLN tracers by a rat model. We also performed histological examinations of the silica coated fluorescent nanoparticles for lymph nodes to clarify the modality of transportation of them by transmission electron microscopy (TEM).

## Results

### Formation of silica coating and concentration of TEOS

**Figure 1:**
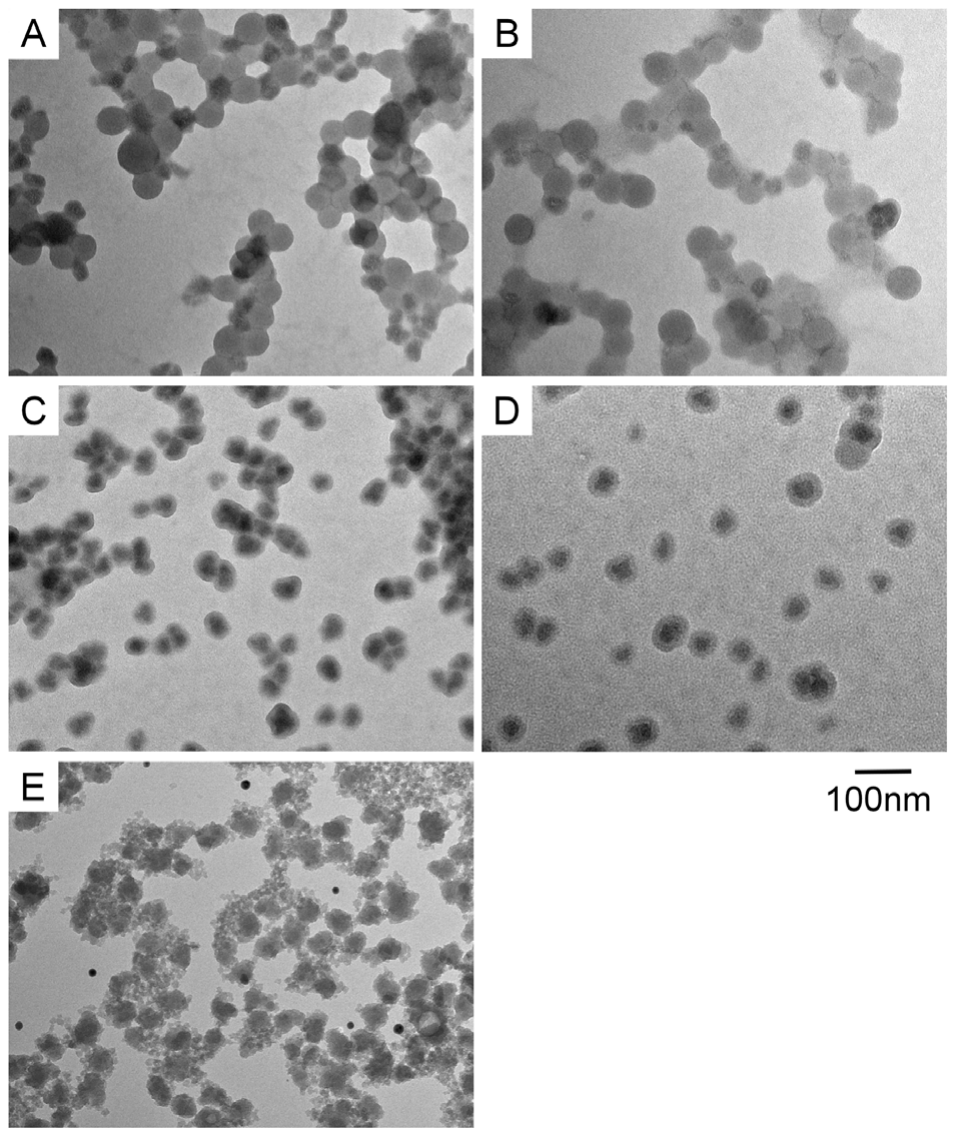
Electron microscopic images of fluorescent nanoparticles with silica coating at various TEOS concentrations. (A) Electron microscopic image of fluorescent nanoparticles and silica formation at 0.00038 M TEOS. (B) Fluorescent nanoparticles and silica formation at 0.0015 M TEOS. (C) Fluorescent nanoparticles and silica formation at 0.009 M TEOS. (D) Fluorescent nanoparticles and silica formation at 0.02 M TEOS. (E) Fluorescent nanoparticles and silica formation at 0.2 M TEOS.

We performed the silica coating with various tetraethoxyorthosilicate (TEOS) concentrations ranging from 0.00038 to 0.2 M. Figure 1A and Figure 1B showed that low TEOS concentration of 0.00038 to 0.0015 M did not evoke formation of silica shell. Figure 1C shows that a TEOS concentration of 0.009 M evoked silica coating, but the nanoparticles aggregated. Figure 1D showed that a TEOS concentration of 0.02 M was optimal for the silica shell formation on 40 nm polystyrene nanoparticles, and Figure 1E showed that a high TEOS concentration (0.2 M) caused the formation of silica nanoclusters and silica coating could not be formed.

**Figure 2:**
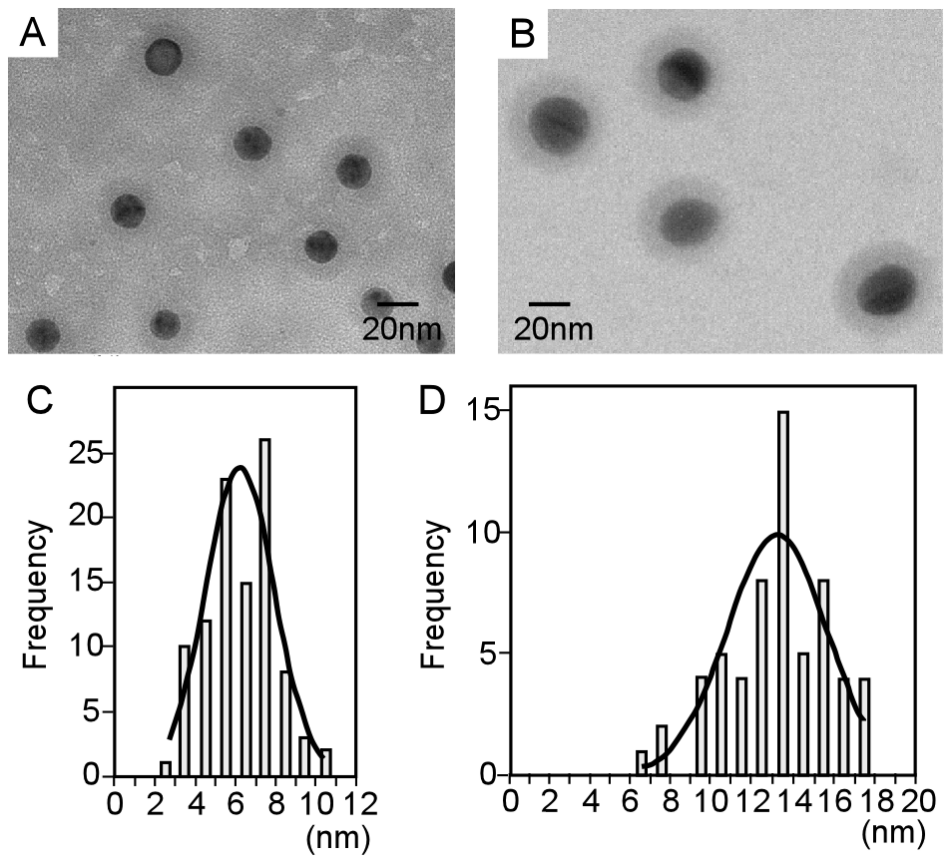
Electron microscopic images and distribution of silica thickness on fluorescent nanoparticles of 20 and 40 nm. (A) Electron microscopic image of 20 nm fluorescent nanoparticles with silica coating. (B) Electron microscopic image of 40 nm fluorescent nanoparticles with silica coating. (C) Distribution of silica coating thickness on 20 nm fluorescent nanoparticles. (D) Distribution of silica coating thickness on 40 nm fluorescent nanoparticles.

The distribution of diameter was shown in Figure 2. The silica shell thickness on 20 nm nanoparticles was 6.3+1.7 nm (Figure 2A) while the thickness of silica shell on 40 nm nanoparticles was 13.2+2.5 nm (Figure 2B). The silica shell thickness of 40 nm coated nanoparticles revealed a single peak while the silica coated 20 nm nanoparticles revealed two peaks in the histogram (Figure 2C and Figure 2D). We employed silica coated 40 nm nanoparticles for measurement of zeta potential and animal experiments on SLN detection.

The dispersion stability of the silica coated fluorescent nanoparticles was also measured by a zeta potential and submicron particle size analyzer (ELS-8000, Otsuka Electronics). The zeta potential of silica coated 40 nm nanoparticles was 22.4 mV at pH 7.2.

### Fluorescence property of the fluorescent nanoparticles

**Figure 3:**
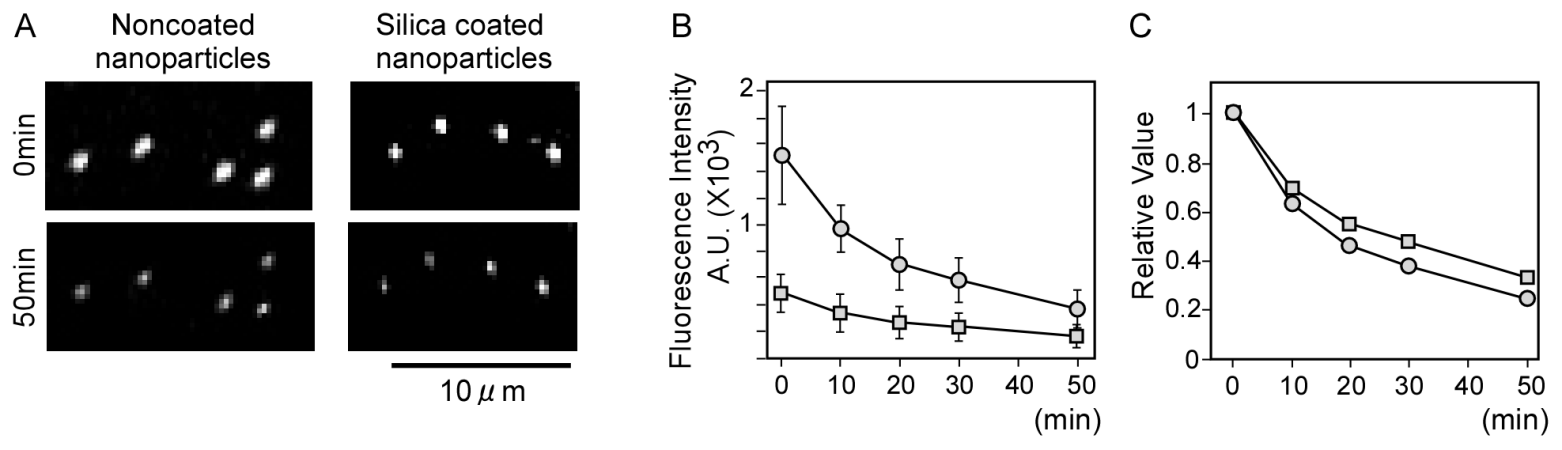
Fluorescence images and intensities of silica coated and noncoated nanoparticles. (A) Fluorescence image of silica coated and noncoated nanoparticles with continuous laser irradiation. (B) Changes in the fluorescence intensity of silica coated and noncoated nanoparticles with continuous laser irradiation. Open circles and squares show noncoated nanoparticles and silica coated nanoparticles, respectively (n = 10). Error bar, s.e.m. (C) Changes in the relative value of fluorescence intensity of silica coated and noncoated nanoparticles in (B). The mean value of fluorescence intensity at 0 min was defined as 1. Open circles and squares show noncoated nanoparticles and silica coated nanoparticles, respectively.

The comparison of the fluorescence property of the 40 nm nanoparticles before and after coating with 0.02 M TEOS is shown in Figure 3. A spectral analysis of the silica coated fluorescent nanoparticles exhibited no significant difference from the original nanoparticles (data not shown).

Florescence stability of noncoated and silica coated nanoparticles was estimated by continuous irradiation of laser light (Figure 3A). Although fluorescence intensity of noncoated nanoparticles was 3-fold higher than silica coated nanoparticles at 0 min (Figure 3B), the light stability of silica coated nanoparticles was 1.3-fold greater than noncoated nanoparticles (Figure 3C, 50 min).

### Fluorescent imaging of the inguinal lymph nodes in rats

**Figure 4:**
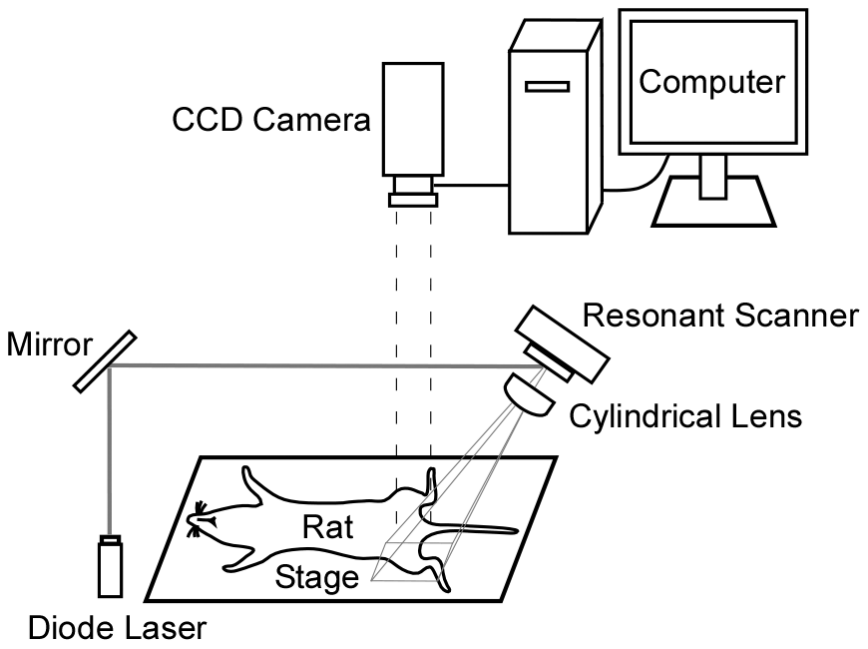
Schematic of fluorescent imaging system. The fluorescent imaging system was comprised of a laser unit, a resonant scanner, a cylindrical lens and a charge-coupled device camera and a computer.

**Figure 5:**
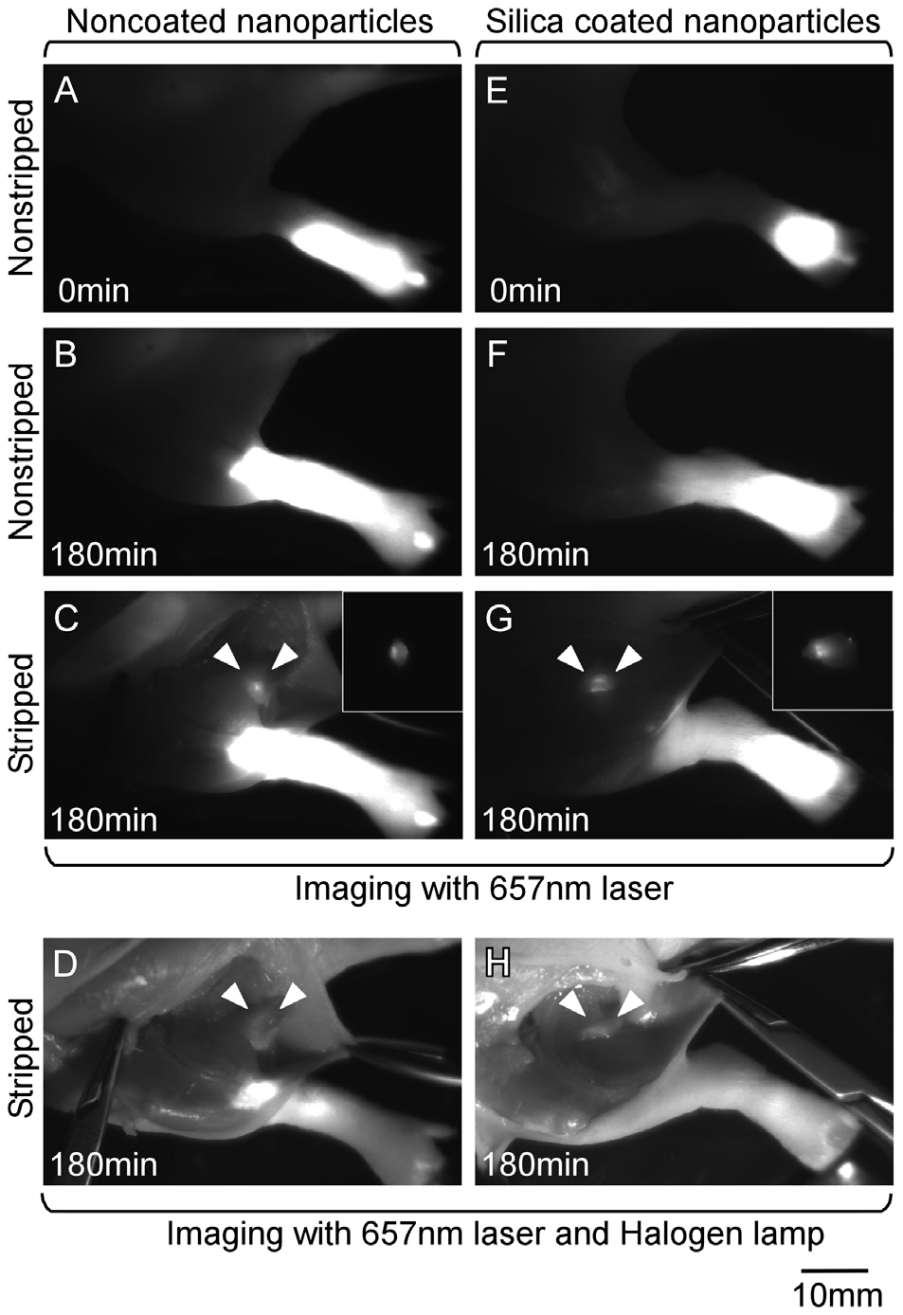
Fluorescence images of sentinel nodes of rat lower extremities by silica coated and noncoated nanoparticles. (A) Fluorescence image at 0 min after injection of noncoated nanoparticles. (B) Fluorescence image at 180 min after injection of noncoated nanoparticles. (C) Fluorescence image at 180 min after injection of noncoated nanoparticles with skin stripping. (D) Image at 180 min under light illumination after injection of noncoated nanoparticles with skin stripping. (E) Fluorescence image at 0 min after injection of silica coated nanoparticles. (F) Fluorescence image at 180 min after injection of silica coated nanoparticles. (G) Fluorescence image at 180 min after injection of silica coated nanoparticles with skin stripping. (H) Image at 180 min under light illumination after injection of silica coated nanoparticles with skin stripping.

The silica coated fluorescent nanoparticles were injected subcutaneously in the feet of rats. The imaging set-up and fluorescent images by silica coated nanoparticles are shown in Figure 4. At 0 min and 180 min after injection, the lymphatic systems were not observed from outside the body for both noncoated and coated nanoparticles (Figure 5A and 5B, and Figure 5E and 5F, respectively). As sentinel lymph nodes of the hind extremities are usually located in inguinal and popliteal regions, we assigned the sentinel node as an emerging fluorescence point after stripping the skin of the inguinal and popliteal regions. The fluorescent lymph nodes could be observed after stripping the skin in both cases with noncoated and coated nanoparticles (Figure 5C and Figure 5G, respectively). Macroscopic images at 180 min after skin stripping under light illumination are showed in Figure 5D and Figure 5H for the cases with noncoated and coated nanoparticles, respectively.

### Histological analysis of the rat inguinal lymph nodes

**Figure 6:**
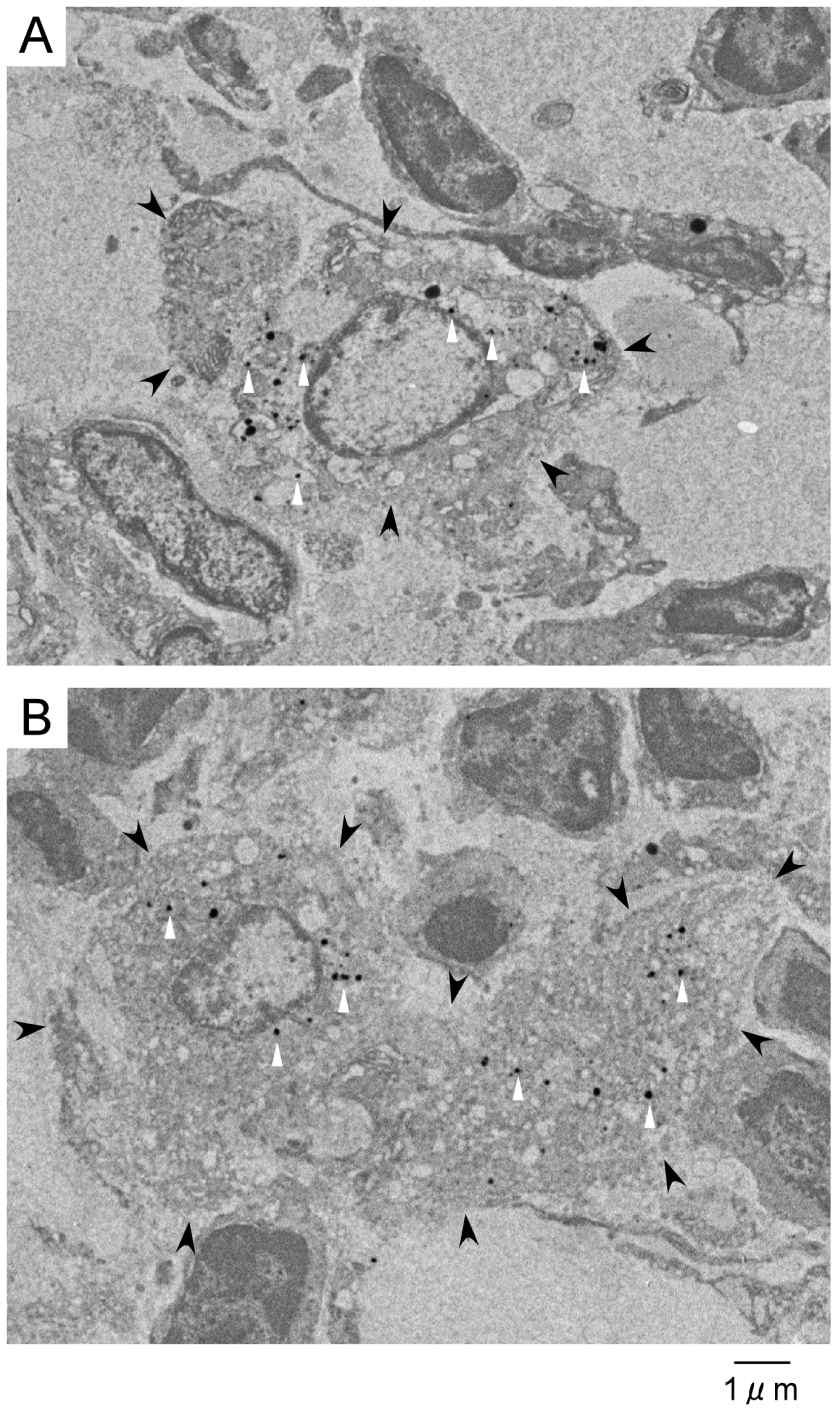
Electron microscopic images of phagocytes in inguinal lymph nodes. (A) A macrophage in inguinal lymph nodes with silica coated fluorescent nanoparticles injection (Black arrow head; the outline of the macrophage, white arrow head; the silica coated fluorescent nanoparticles). (B) A dendritic cell in inguinal lymph nodes with silica coated fluorescent nanoparticles (Black arrow head; the outline of the dendritic cell, white arrow head; the silica coated fluorescent nanoparticles).

We observed inguinal lymph nodes by TEM (Figure 6A and Figure 6B). The inguinal lymph nodes were fixed by formalin and histological examinations were performed. The phagocytes of macrophages and dendritic cells were distinguished by their morphological features. Macrophages are polyhedral free reticulum cells with many pseudopods around the cell membrane. Dendritic cells are satellite-shaped tissue-specific reticulum cells with long radially extending processes. Figure 6A and Figure 6B are macrophages and dendritic cells with nanoparticles injection, respectively. The silica coated fluorescent nanoparticles were observed to be deposited in both the macrophages and dendritic cells by TEM (Figure 6A and Figure 6B) and fluorescence (images not shown).

## Discussion

In this study, we used the modified Stöber method to construct the silica coating on fluorescent nanoparticles in size of 20 and 40 nm. Under the low concentration of the TEOS (0.00038 to 0.009 M), the silica layer was not formed on the polystyrene particles. Under the optimal concentration of the TEOS at 0.02 M, the silica shells were formed and the particles did not aggregate. The thickness of the silica layer is homogenous and well controlled by this technique. More fine modification of TEOS around 0.02 M may lead to fine control of the shell thickness and diameter.

Nanoparticles for medical application should be stabilized by modification with hydrophilic or hydrophobic molecules in order to prevent aggregation in water or organic solvent, respectively [15]. In our study, the concentration of TEOS of 0.00038 to 0.009 M did not work for the silica coating of the fluorescent nanoparticles and resulted in the deposition of gel-like silica particulates around the fluorescent nanoparticles.

In clinical practice using the dye method to identify the SLN, the incision should be made within a few minutes since the dye moves instantaneously from the injection site to the lymphatic system. Moreover, if the lymph vessel is damaged by skin incision before dye moves to SLN, the lymph node will not be dyed. Furthermore, when a prolonged time is spent for detecting the SLN, many non-sentinel lymph nodes will also be dyed [16]. Previous studies have shown that large nanoparticles over 100 nm were not optimal for the detection of the SLN because they require so long period for movement to the SLN, and the ideal size was determined to be 40 nm among 20, 40, 100 and 200 nm [8]. The ideal SLN tracer arrives at a lymph node quickly and moves out from the lymph node slowly. Fluorescent nanoparticles are good candidates for such ideal tracers [17]–[21]. In this study, we observed the inguinal lymph nodes from 0 to 180 min after injection. The silica coated fluorescent nanoparticles moved to these lymph nodes *via* the lymphatic network under the skin.

The durability in enhancement of the silica coated 40 nm fluorescent nanoparticles was about 5 hr or more (data not shown). Use of near infrared avoids autofluorescence and improves the signal to noise ratio. The wavelength of near infrared is ideal for fluorescence measurement of the biological system in consideration with the autofluorescence spectrum [8].

Physical stability is an advantage of the silica coating and silica shell protected the inner polystyrene nanoparticles in the tissue processing for the microscopic and TEM observations. It is important to know the impact of the material on organisms and *in vivo* dynamics to realize clinical applications. Silica coated fluorescent naonparticles could be tracked by both macroscopic fluorescence measurement and microscopic TEM observation. The translocation of a nanoparticle to the lymph vessels and the uptake to a lymph node depends upon the size of nanoparticles. Nanoparticles of small size are directly conveyed to a lymph node by the physical active transport of lymphatic flow. However, large-sized nanoparticles are passively conveyed by macrophages after phagocytosis.

The silica coated nanoparticles maintained enhancement for several hours. Prolonged enhancement is explained by phagocytosis of fluorescent nanoparticles by macrophages and dendritic cells in lymph nodes as shown in Figure 6. There has been no evidence identifying the cause of the prolonged enhancement of fluorescent nanoparticles since existing fluorescent polystyrene nanoparticles are easily broken in the process of sample preparation for TEM. We demonstrated, for the first time, the presence of fluorescent nanoparticles in macrophages and dendritic cells by TEM.

Although we used near infrared based on the optical traits of the organism to improve sensitivity, the limit for detection is less than 1 cm depth. Novel technologies are also expected for detecting fluorescence in deep sites of organs like the acousto-optic modulation imaging for realizing the fluorescence measurements in the SLNB [22].

## Materials and Methods

### Fluorescent nanoparticles

FluoSpheres® (Invitrogen) are commercially available fluorescent polystyrene nanoparticles containing fluorescent molecules within them. There are variations in the fluorescence wavelength (515 to 755 nm) and the particle size (20 to 1000 nm). The 20 nm and 40 nm fluorescent nanoparticles (F-8783, 2% solids and F-8789, 5% solids), with excitation/emission wavelengths of 660 nm/680 nm, were used for this experiment.

### Reagents for the Silica Coating

The chemicals of polyvinylpyrrolidone (PVP), ethanol (95.5%), tetraethoxyorthosilicate (TEOS, 95%) and ammonia (NH_4_OH, 25% aqueous solution) were obtained from Wako Pure Chemical Industries. Ultrapure deionized water (resistivity higher than 18 MΩ cm) was used in the preparations for the silica coating.

### Silica Coating Procedures

The silica coatings of fluorescent nanoparticles were performed in a hermetically sealed reactor equipped and the total volume of the reactor was 30 ml. We performed silica coating by dissolving PVP (0.3 g) in the water (5.571 ml) and then continuously agitated the solution with a magnetic stirrer until the complete dissolution of PVP. The PVP solution was vigorously stirred after the addition of fluorescent polystyrene nanoparticles (0.1 ml). Ethanol was subsequently added to the PVP/nanoparticles mixture and a pure silica precursor TEOS was added to the PVP/nanoparticles/ethanol mixture. We used ethanol at volumes of 23.89, 23.88, 23.80, 23.75 and 22.48 ml for the concentrations of the TEOS of 0.00038, 0.0015, 0.009, 0.02 and 0.2 M in order to find the appropriate concentration for silica shell formation, respectively. Ammonia (0.42 ml) was added to the PVP/nanoparticles/ethanol/TEOS mixture, followed by the incubation of the mixture at room temperature for 12 hr or more. The concentration of PVP, water and ammonia of the mixture were 10 g/l, 10.9 M and 0.4 M, respectively. And the volume of the mixture was 30 ml. The silica coated fluorescent nanoparticles were concentrated by a rotary evaporator (NE-1, Tokyo Rikakikai) and then were rinsed by a centrifuge (CP 70MX, Hitachi). The final volume of the silica coated fluorescent nanoparticle suspension was 10 ml.

### Electron Microscopy

The silica coated fluorescent nanoparticles suspension was directly trickled onto the collodion membrane attachment mesh (Nisshin EM) and images were observed using a TEM (H-7600, Hitachi Science Systems). The TEM was operated at an 80–100 kV accelerating voltage.

### Evaluation of fluorescence intensity

Fluorescence intensity of individual silica coated nanoparticles was estimated by a specially designed fluorescence measurement system. The system was composed of a fluorescent microscope (IX71, Olympus), an electron multiplying charge-coupled device camera (iXon^EM^+ DU-897, Andor), a confocal unit with a Nipkow disc (CSU10, Yokogawa) and control computing system as noted in our previous study [23].

### Preparation of the tissue sample

Tissue specimens were fixed and immobilized in 2% glutaraldehyde buffered with 0.1 M sodium cacodylate buffer solution (pH 7.4). The tissue was washed with 3 changes of 0.1 M sodium cacodylate buffer (pH 7.4) and postfixed for 120 min in a solution of 1% OsO4 buffered with 0.1 M sodium cacodylate (pH 7.4), then washed in three changes of deionized water and dehydrated in 50% to 100% ethanol. Propyleneoxide was used as a transitional solvent. The tissue was infiltrated overnight in a 1∶1 mixture of Epon-Araldite and propylene oxide. On the following day, the 1∶1 mixture of Epon-Araldite and propylene oxide was removed and replaced with 100% Epon-Araldite. The tissue specimens were infiltrated with resin for 8 hr and polymerized for 48 hr at 60°C. The sentinel node tissue was embedded in a resin and cut into 60 nm thin sections by ultramicrotome (Ultracut S, Leica Microsystems). Thereafter, TEM observations were performed.

### Instrumentation for fluorescence lymph node detection

A laser scanning fluorescence imaging system consisted of a laser unit, a resonant scanner (2K01, Optron) which resonant frequency is 200 Hz, a cylindrical lens and a charge-coupled device (CCD) camera (ORCA II-ER, Hamamatsu Photonics). A diode laser (wavelength 657 nm, 56ICS153/HS, Melles Griot) was used for excitation. The inguinal and femoral areas were continuously scanned over an area of 30×50 mm. The fluorescence images were observed using a CCD camera with a band-pass filter (filter confer wavelength/680 nm, with full width at half maximum/30 mm) (Figure 4).

### Animal Experiments

Male Donryu rats, 5–7 weeks old and 150–170 g in weight (Charles River Laboratories Japan) were used. They were anesthetized with pentobarbital sodium (20 mg/kg) by injection into the abdominal cavity. The hair on the lower half of the body was shaved to avoid autofluorescence. The rats were then fixed on a sample table in supine positions and the silica coated fluorescent nanoparticles suspensions of 50 µl were injected in the foot pad of the hind leg. After observation through the skin for 180 min, the skin was stripped off and the SLN were removed. Histological observations by TEM were performed to detect silica coated nanoparticles in the lymph nodes.

These experiments were carried out based on the Tohoku University guidelines for animal experiments, after permission was granted by the Committee of Animal Use and Care of the Tohoku University. The approval number of these experiments was 21MdA-72.
